# Sub–100 nm Nanoparticle Upconcentration in Flow by Dielectrophoretic Forces

**DOI:** 10.3390/mi13060866

**Published:** 2022-05-30

**Authors:** Maria Dimaki, Mark Holm Olsen, Noemi Rozlosnik, Winnie E. Svendsen

**Affiliations:** 1 DTU Bioengineering, Technical University of Denmark, Søltofts Plads, Bldg 221, 2800 Kongens Lyngby, Denmark; wisv@dtu.dk; 2 Center for Plastics and Packaging Technology, Teknologisk Institut, Gregersensvej 1, 2630 Taastrup, Denmark; mols@teknologisk.dk; 3 Eir Diagnostics ApS, Muslingevej 36B, 8250 Egå, Denmark; nr@eir-diagnostics.com

**Keywords:** dielectrophoresis, upconcentration, microfluidics, nanoparticles

## Abstract

This paper presents a novel microfluidic chip for upconcentration of sub–100 nm nanoparticles in a flow using electrical forces generated by a DC or AC field. Two electrode designs were optimized using COMSOL Multiphysics and tested using particles with sizes as low as 47 nm. We show how inclined electrodes with a zig-zag three-tooth configuration in a channel of 20 µm width are the ones generating the highest gradient and therefore the largest force. The design, based on AC dielectrophoresis, was shown to upconcentrate sub–100 nm particles by a factor of 11 using a flow rate of 2–25 µL/h. We present theoretical and experimental results and discuss how the chip design can easily be massively parallelized in order to increase throughput by a factor of at least 1250.

## 1. Introduction

The separation of subpopulations of particles is important for many biological, diagnostic, or environmental applications. Such particles can be found in a variety of different fluids, including but not limited to drinking water, surface water, blood, serum, urine, and milk, and in sizes ranging from a few tens of nanometers (e.g., viruses, exosomes, liposomes) up to a few tens of micrometers (e.g., blood cells, bacteria). Some particles are available in large quantities in a sample and can therefore be readily identified and processed, while others are very rare, requiring one or several upconcentration steps for their isolation and identification. While exosomes for example are found in concentrations of about 1 × 10^8^ exosomes/mL in blood [1], circulating tumor cells (CTCs) are only present in concentrations of 1–100 cells/mL [2].

Manipulation of micro- and nanoparticles by passive or active techniques has been the subject of research for at least two decades. Microfluidic technology has been the driving force, particularly due to low sample volume requirements, which are very attractive for many diagnostic applications. Properties such as precise control of flow and the relative ease by which externally applied forces can be integrated into these systems make microfluidic technology ideal for most such applications.

Passive techniques utilize the properties of the flow itself along with geometrical modifications to manipulate particles. Several examples of passive systems can be found in the literature. For example, exosomes (size of 30–200 nm) were sorted with a purity of 92% and a recovery of 81% using a combination of inertial microfluidics and viscoelastic fluids [3]. Inertial microfluidics by itself is a method that has been used for the size sorting of particles in relatively large microfluidic channels and at relatively high Reynolds numbers [4]. Although the method is powerful for the size separation of particles, it operates mostly on particles in the micrometer range. The use of deterministic lateral displacement arrays (DLDs) is another popular passive method for particle separation that can be applied to particles from 20 nm to several tens of micrometers. However, for nanoparticle sorting, it is also necessary to use pillars in the nanometer range, which complicates fabrication and use of the system, and the method is also limited by throughput [5].

For continuous nanoparticle separation, active techniques are the preferred method. Here microfluidics is combined with some sort of external force that acts on the particle and displaces it away from its initial position. External forces are mainly electrical, magnetic, acoustic, or optical. Most of these forces scale with the particle size, which means that they are generally very small for nanoparticles, and have limited throughput [6]. Magnetic forces require the particle to be sorted to either have magnetic properties or be labeled with magnetic particles.

One of the most popular active manipulation methods is the use of electrical forces generated by electrodes immersed in a liquid. Electrical forces acting on uncharged but polarizable particles give rise to a so-called dielectrophoretic (DEP) force. This force depends on particle volume and on the electrical properties of the particles and the medium they are dispersed in (see Section 2.1 for further details). The method requires rather high voltages, particularly in the case where the so-called insulator DEP is used [7]; the high voltages lead to Joule heating and electrothermal flow that can disturb the separation [8].

The sorting of different biological particles, particularly cells, in a continuous flow by DEP is very well studied. One of the main obstacles is the similarity between human cells; they have similar densities and similar turnover frequencies [9]. Therefore, developed systems utilize long channels, multifrequency DEP, and pretreatment protocols to achieve separation. The method is quite efficient for this type of particles because they are rather large; DEP forces scale with the particle radius, and cells are of the order of 5–30 µm.

The DEP force is also highly localized, as it is proportional to the electric field gradient, which declines rapidly the further away from the electrodes the particle is. Therefore, much effort has been put into designing electrodes that can generate significant gradients in as much of the volume of a microfluidic channel as possible, where the DEP force should act. These geometries include top and bottom electrodes; sidewall electrodes, also called 3D electrodes [10]; liquid electrodes, where the channel geometry results in virtual sidewall electrodes, even though the real electrodes are planar [11]; and other techniques, such as nanosized orifices [12], laser-induced DEP [13], and nanopipette-based DEP [14]. Characteristic of all the above is a need for a specialized fabrication process or additional equipment to achieve the large gradients necessary to affect the movement of nanoparticles.

In this paper, we present a simple and scalable microfluidic chip with microsized electrodes generating the electrical field for the continuous sorting of sub–100 nm particles, with the aim of upconcentrating these for further applications. Apart from an AC voltage generator at a single frequency, no other equipment is required for its function, and the fabrication process is simple and modifiable for large-scale production using screen printing and injection molding. We used numerical simulations to find an optimal electrode design and tested the chip using particles with sizes as low as 47 nm, corresponding to the size of small viruses. We further show how this simple design is easily scalable for increasing throughput.

## 2. Materials and Methods

### 2.1. General Requirements

The main function of the chip is to concentrate sub-nanometer particles at the center of the channel using dielectrophoretic forces generated by a set of electrodes powered by an AC voltage. The time-averaged dielectrophoretic force is given by Equation (1) for a spherical particle of radius α [15].

(1) $$F_{DEP}=\pi \cdot \;\alpha^{3}\cdot \varepsilon_{m}\cdot \varepsilon_{0}\cdot Re\left(\frac{\varepsilon_{p}^{*}-\varepsilon_{m}^{*}}{\varepsilon_{p}^{*}+2\varepsilon_{m}^{*}}\right)\cdot \nabla \left|E^{2}\right|$$

where $\varepsilon_{m}$ is the relative permittivity of the suspending medium, $\varepsilon_{0}$ is the permittivity of free space, and E is the applied electric field amplitude. The quantities $\varepsilon_{i}^{*}$, where the subscript *i* can be either *p* or *m*, refer to the complex permittivities of the particle (*p*) and the medium (*m*). These are given by Equation (2):

(2) $$\varepsilon_{i}^{*}=\varepsilon_{0}\varepsilon -j\frac{\sigma }{\omega }$$

where σ is the conductivity, ε is the relative permittivity, and ω is the angular frequency of the applied electric field.

The term containing the complex permittivities is known as the Clausius–Mossotti factor and determines whether the particles are attracted towards the electrodes (known as positive DEP) or move away from them (negative DEP). Due to surface conductance effects, polystyrene beads have been shown to exhibit positive DEP at frequencies below 1–10 MHz [16].

**Figure 1:**
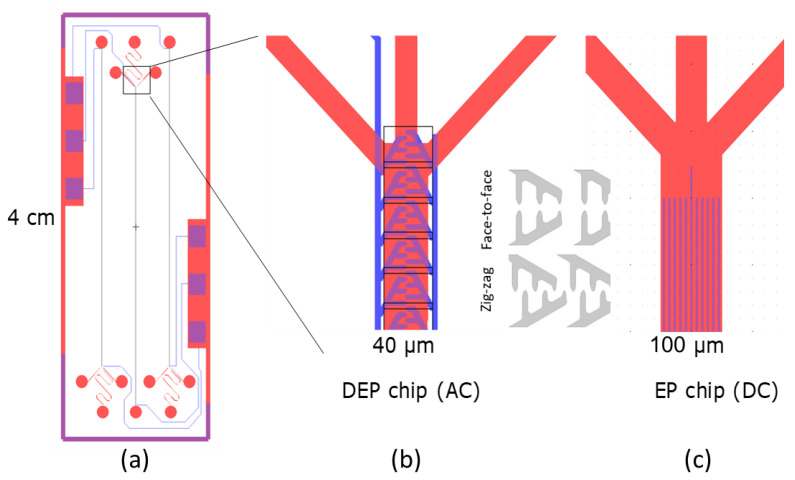
(**a**) The general chip design. (**b**) The channel contains a single channel with electrodes at the bottom. In the case of the AC chip, the electrodes are either in a zig-zag or face-to-face configuration (inset). (**c**) In the case of the DC chip, the electrodes are simple lines running parallel to the channel length.

The term $\nabla \left|E^{2}\right|$ in Equation (1) is dependent on the applied voltage (*V*) and the distance between the electrodes (*d*). The quantity can be approximated as V2d3, which means that the force is essentially inversely proportional to the distance between the electrodes to the power of 3. A small electrode distance will generate a localized large field at the tip of the electrodes, but nowhere else in the channel, unless the electrodes are designed in such a way so that they maintain a small spacing at a larger proportion of the channel width, without compromising the inhomogeneity of the electric field. Moreover, to achieve a large field across the channel height, we would ideally need electrodes on both the channel top and bottom. This, however, significantly complicates the fabrication process. Therefore, electrodes were placed only at the bottom of the channel, and the channel height was restricted to 25 µm. The channel width was restricted to 40 µm, for the same reasons. The general chip design is shown in Figure 1a. Each chip contains three individually addressed electrode arrays inside a microfluidic channel. There is one inlet and three outlets for each channel, and the nanoparticles are collected through the central outlet after they have been focused by the DEP forces. A close-up of the outlet region is shown in Figure 1b. Electrodes were placed either directly opposite each other, or in a zig-zag pattern. The angles of the main electrodes were at 60 degrees relative to the channel middle, and the protrusions were spaced 5 µm (face-to-face) and 6 µm (zig-zag) apart, with opposite electrodes having a minimum distance of 2.5 µm (face-to-face) and 2 µm (zig-zag).

Further, the option of using a DC voltage for the same purpose was investigated. In this case, a voltage was applied on a thin electrode running along the entire length of the channel exactly in the middle while several parallel ones provided the grounding voltage. The channel was 100 µm wide, the electrodes were 3 µm wide, and the distance between them was 6 µm. The particles were attracted towards the middle electrode due to electrophoresis, as they are charged. The general design for the DC chip is shown in Figure 1c. The dimensions of the two chips (AC and DC) were the result of a numerical optimization of the upconcentration performance under AC or DC conditions.

Each chip contained 3 cm long channels with electrodes on the bottom. The main channel splits into the three outlets, with the middle outlet designed to carry about 10% of the flow by making it longer than the outer outlets. All three outlets of the DC chips were 50 µm in width, while all three outlets of the AC chips were 20 µm in width. As all the particles coming from the inlet are concentrated in the middle of the channel, the middle outlet will collect all the particles but only in a 10th of the original fluid volume, thus resulting in the upconcentration of the sub-nanometer particles. Chips with more than one row of electrodes were also fabricated in order to evaluate the ability of the process to create identical electrodes along a large wafer surface.

### 2.2. Numerical Simulations

COMSOL Multiphysics Version 5.4 was used to calculate the particle trajectories due to the flow and the dielectrophoretic force. To minimize the computation time, only a 500 µm long channel was simulated. The electric field gradient and the dielectrophoretic force were calculated using the AC/DC module, while the flow profile was calculated using the laminar flow module. A voltage of 20 V peak-to-peak was applied to the electrodes in order to calculate the electric field. All other boundaries were set to the default boundary condition of “Electric Insulation” (n·J=0). For the flow simulations, all boundaries apart from the inlet and outlet were set with a no-slip boundary condition. A flow rate of 1 µL/h was applied to the inlet, and a pressure of 0 was used as a boundary condition at the outlet. Once the flow profile and the electric field were calculated, the particle tracing module was used to calculate the trajectories of particles of varying diameters (in the range of 30–100 nm) and a density of 1200 kg/m^3^ starting at the channel inlet on a line spanning the channel width at half the channel height. The particles were affected by the flow, gravity (which was, however, negligible for nanoparticles), and dielectrophoresis. The general size of the mesh was set to “coarse”, calibrated for fluid dynamics; however, all boundaries were set to a “normal” size, and a mesh control face was added in the middle of the channel (xz plane running along the channel length) with a maximum element size of 2 µm.

**Figure 2:**
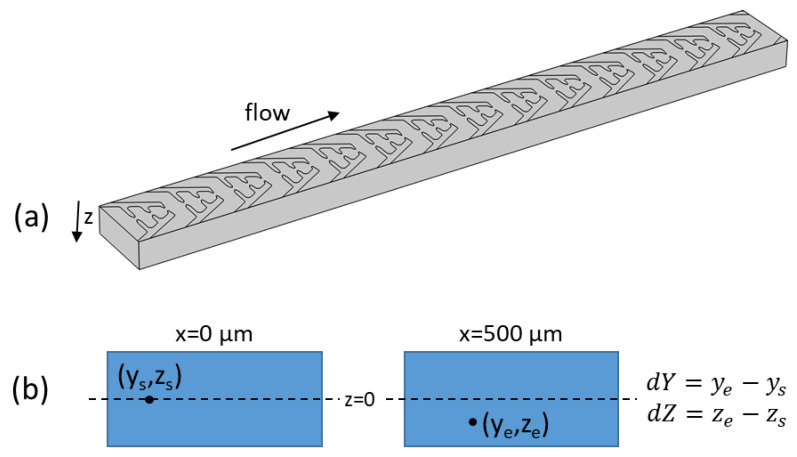
(**a**) The 500 µm long channel with electrodes on the bottom side used for the simulations. (**b**) The starting and ending position of each particle starting at a certain (y,z) point at x = 0 µm is noted after the particle has moved in the channel to x = 500 µm. The displacement along the height and width is used to evaluate how well each electrode design is performing in terms of particle focusing.

The particles starting and ending positions on the y and z axes (Figure 2) of a 500 µm long channel were noted, and the displacement was used to evaluate the performance of each design. Due to computational constraints, Brownian motion was not included in the COMSOL simulations; however, the values of the velocity and electric field in a unit cell of the geometry were exported in matrix form to Matlab (Version 2017b), and an algorithm was developed to calculate the particle trajectories with the thermal force included [17]. The algorithm stops when the particle touches one of the boundaries (sidewalls of the channel or bottom). Due to the complexity of the simulation, bouncing on the boundaries has not been included. The Matlab algorithm can be performed for any flow velocity (by correcting the COMSOL calculated velocity with the correct multiplication factor, MF_vel = (wanted flow rate)/(COMSOL used flow rate). Similarly, the dielectrophoretic force can also be adjusted in the Matlab program by a multiplication factor MFforce = (wanted voltage)^2^/(COMSOL used voltage)^2^.

The simulations using Matlab were performed with an applied AC voltage of 20 V peak-to-peak and for particles 100 nm in diameter. The particle movement was simulated for flow velocities of 1 µL/h, 10 µL/h, and 25 µL/h. To gain statistical information, 10 to 200 particles were simulated from each position. For the end position graphs, only those particles that had ended their movement on the bottom boundary were included.

We also performed similar simulations for the DC chip (data not shown), which resulted in the chosen chip design and experimental conditions.

### 2.3. Chip Fabrication Process

The two most promising designs were fabricated using standard photolithography processes on silicon wafers. The fabrication was completed at the National Center for Nanofabrication and Characterization and the Technical University of Denmark. Briefly, 200 nm of silicon oxide was thermally grown on a standard 4-inch silicon wafer with a thickness of 525 µm. Then, 1.5 µm of AZ5214E resist was spun on the wafer and exposed to UV light (365 nm) for 7 s (KS Aligner) with a power of 8 mW/cm^2^ through a mask containing the electrodes in hard contact mode. The resist was developed using a standard AZ resist developer (AZ351B). Then, 10 nm of Ti and 100 nm of gold were evaporated on the wafer by e-beam evaporation using a Wordentec QCL 800. This was followed by a lift-off process in acetone for 20 min. SU8-2075 was spun on the wafer and exposed through a second mask containing the microfluidic channels using the KS Aligner. The target height for the SU8 resist was 20 µm. After development in propylene glycol methyl ether acetate (PGMEA) for 10 min, the chips were diced using a diamond saw.

### 2.4. Measurement Setup

A holder was fabricated using micromilling. The holder contained the silicon chip, a PDMS gasket to close the microfluidic channels, and holes for the inlet and outlet tubing and the electrical connections using spring pins.

An AC signal generator (TG2000, Aim and Thurlby Thandar Instruments, Cambridgeshire, UK) was used to apply an AC voltage of varying amplitude and frequency to the electrodes. Voltages from 1 V to 20 V peak-to-peak were used at a frequency of 200 kHz. This frequency was selected after evaluating the performance of the particle focusing at frequencies ranging from 10 kH to 1 MHz. The flow rate was 2 µL/h. To avoid the particles becoming stuck to the middle electrode, an on–off frequency of 3 Hz was used, although frequencies between 1 Hz and 5 Hz were also investigated.

For the DC case, a DC voltage of 2 V was used. The flow rate in this case was varied between 10 and 50 µL/h. A standard syringe pump (Chemyx Fusion 200) was used for controlling the flow rate.

Polystyrene beads of sizes ranging between 47 nm and 1 µm were used. The beads were fluorescently labeled (Fluoresbrite YG Microspheres, from Polysciences, Inc., Warrington, PA, USA). The bead movement was observed using a fluorescent microscope (Nikon TE2000U). The beads were suspended in tap water with an addition of 0.1% Tween (Sigma Aldrich, St. Louis, MO, USA). The pH of the solution was 7 and the conductivity was 50 mS/m.

### 2.5. Result Evaluation

ImageJ was used to measure the minimum distance between electrodes for the AC chip, as well as the dimensions of the electrodes in the DC chip. Image J was also used in order to evaluate whether the fluidic channels were designed properly so that only 10% of the flow was collected in the central channel. In this case, recordings of the particle movements were taken, enhanced, and analyzed by manually tracking the position of up to 20 particles per outlet channel. The average velocity of each particle was calculated, and from these results, an average velocity for each outlet was found. The concentration ability of the fluidic system was then calculated as follows:

(3) $$\mathrm{Ratio}=\frac{\mathrm{Middle}\;\mathrm{channel}\;\mathrm{velocity}}{\mathrm{Upper}+\mathrm{Middle}+\mathrm{Lower}\;\mathrm{channel}\;\mathrm{velocity}}$$

**Figure 3:**
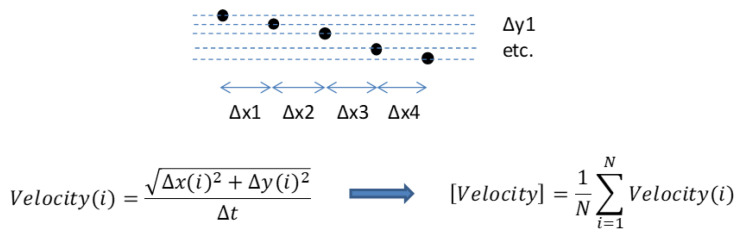
The general algorithm for the calculation of the particle velocity by using recordings of particle movement in the channel. To calculate the average velocity of each particle, 5 to 30 positions were tracked per particle.

Figure 3 outlines the general algorithm of the velocity calculation for each particle.

## 3. Results

The results of the chip fabrication, the simulations, and the experimental particle upconcentration are presented in this section.

### 3.1. Chip Fabrication

**Figure 4:**
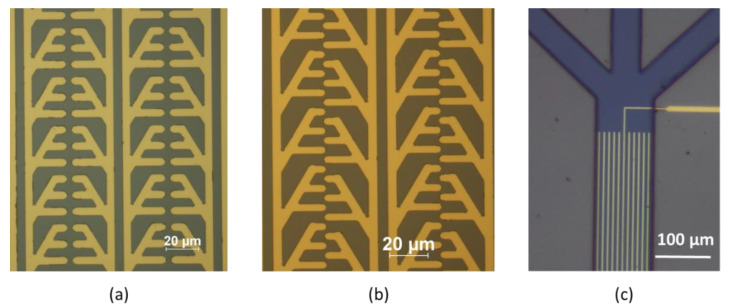
Close up of the chip. (**a**) Face-to-face electrode design. (**b**) Zig-zag electrode design. (**c**) DC chip design.

A close-up of the electrodes and SU8 channels can be seen in Figure 4.

The lift-off process results in well-defined structures. ImageJ was used to measure the distances between the electrode tips in both designs. For the face-to-face design, the mean distance between the electrode tips is 2.53 ± 0.10 µm, while for the zig-zag structure, the minimum distance between the electrode tips is 1.79 ± 0.18 µm. The DC electrodes are 3.24 ± 0.14 µm. All results are based on 10 different measurements.

### 3.2. Numerical Simulations

**Figure 5:**
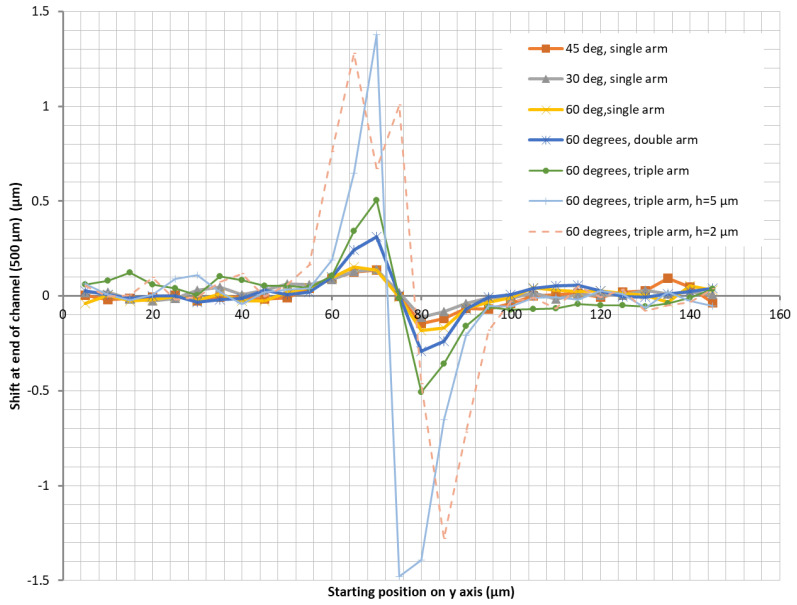
Particle displacement along the channel width after 500 µm of channel length under the influence of dielectrophoretic forces. Unless otherwise indicated, the channel height is 20 µm. The figure shows that the displacement is larger for smaller but unrealistic channel heights. Double refers to electrodes having only two “teeth” instead of the three shown in Figure 1b, which in this graph is indicated as “triple”.

The chip’s geometrical requirements presented in Section 2.1 were based on numerical simulations performed on a much wider channel (150 µm) and with electrodes at angles of 30, 45, and 60 degrees relative to the direction of the channel width, located on the bottom and top of the channel. The results of these simulations, performed for particles of 30 nm in diameter, in a channel with a flow of 1 µL/h, and with single, double, or triple face-to-face electrodes per electrode arm, plotted as described in Section 2.2 and Figure 2 (for the case of a triple electrode), are shown in Figure 5. The graphs show that only the particles situated in the central 40–60 µm of the channel width are influenced by the electric field and that electrodes at a 60-degree angle result in a larger displacement than those at 30 or 45 degrees. We can also see that as the channel height becomes smaller (plotted here for h = 5 µm and h = 2 µm), the displacement becomes bigger. However, channel heights below 10 µm are difficult to fabricate and operate without clogging issues, so this solution was not considered as optimal or realistic.

**Figure 6:**
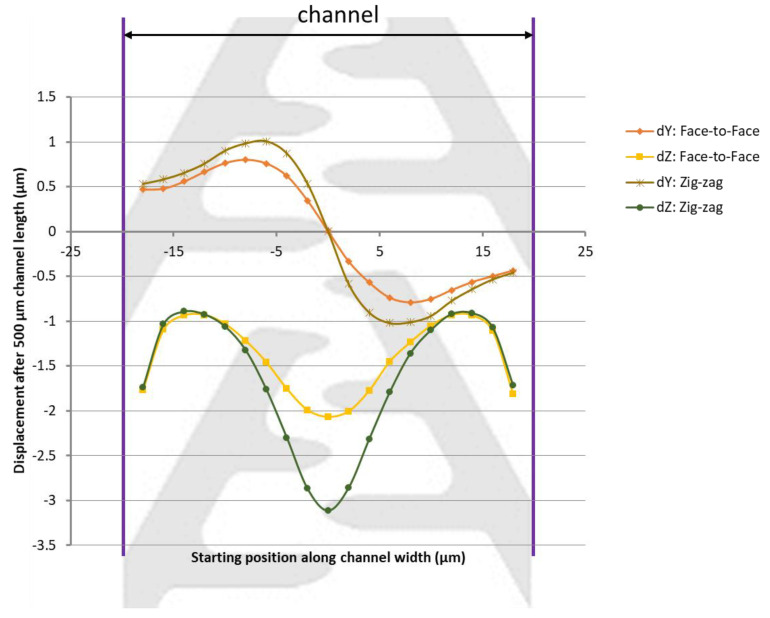
Particle displacement along the channel width (dY) and the channel height (dZ) after 500 µm of channel length under the influence of DEP forces.

Therefore further simulations were conducted, where the channel width was reduced to 40 µm and only a 60-degree angle was used. Furthermore, the particle diameter was increased to 40 nm. The results are plotted in Figure 6, where not only the displacement along the channel width, but also the displacement along the channel height is shown, for both the face-to-face and the zig-zag designs. The zig-zag design is used to illustrate the placement of the electrodes, for the purpose of guiding the eye. The simulations shown were performed for both types of electrodes.

The results show that the zig-zag pattern is more effective than the face-to-face pattern and that there is a displacement of particles regardless of their position along the channel width. The maximum displacement per 500 µm channel length is 1 µm towards the channel center and about 3 µm towards the channel bottom for the zig-zag pattern.

**Figure 7:**
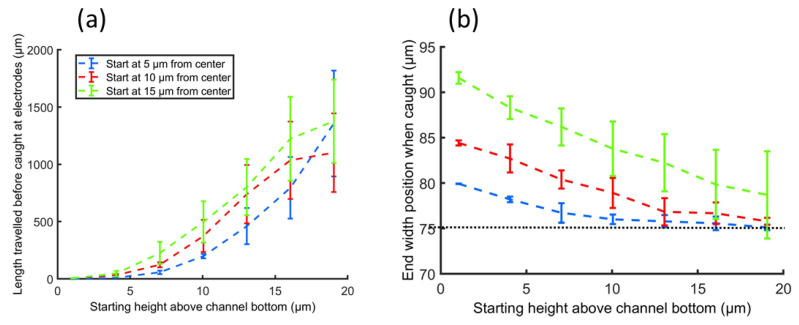
(**a**) The required channel length to catch particles on the electrode plane as a function of starting position along the height of the channel for three different starting positions along the channel width. (**b**) The end position along the width of the channel as a function of starting position along the channel height.

Figure 7 shows the required length to catch a particle (Figure 7a) as well as the position across the channel width where the particle was caught (Figure 7b). It can be seen that particles starting closest to the electrodes (in height) are reaching the channel bottom furthest away from the center of the channel (dotted line at y = 75 µm). This simulation was performed for three different starting positions across the width axis and for a flow velocity of 1 µL/h.

**Figure 8:**
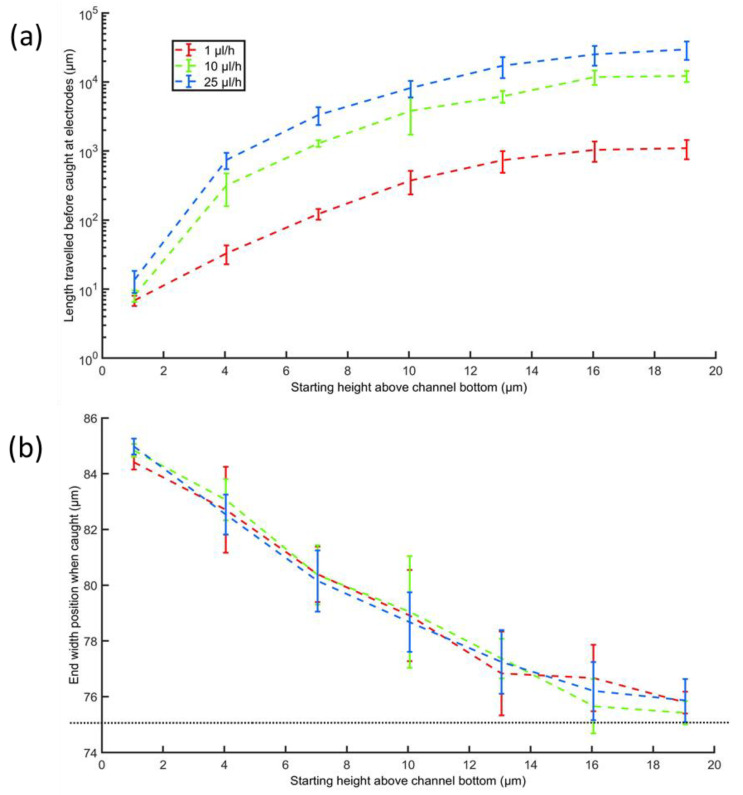
(**a**) Required channel length for catching the particles on the electrodes. (**b**) End position on the width axis. The channel middle is at y = 75 µm (dotted line).

To show the effect of the flow rate on the required channel length and final position, we repeat the simulations for the middle case, i.e., for particles starting 10 µm from the channel center (along the width axis), for three different flow velocities (shown in Figure 8). This middle case shows the worst-case scenario for particles situated in the central 50% of the channel width.

### 3.3. Particle Focusing

**Figure 9:**
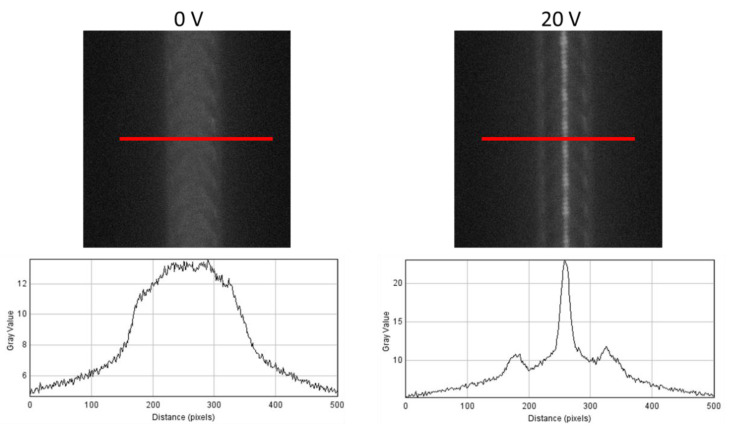
Particle focusing at 20 V peak-to-peak and a frequency of 200 kHz.

As shown in Figure 9, 47 nm particles were successfully aligned using an AC voltage of 20 V peak-to-peak at a frequency of 200 kHz. When the voltage was turned off, the fluorescence was seen in the entire width of the channel, as shown in the signal intensity curve. However, when the voltage was turned on, there was a clear peak in the middle of the channel, indicating that the particles are focused in the channel center as expected.

We observed that the particles tend to stick to the electrodes when they come into contact with them. In order to avoid this effect, we tested periodically switching the frequency on and off with a frequency between 1 Hz and 5 Hz. The results show that frequencies above 5 Hz reduce the focusing efficiency; therefore, a frequency of 3 Hz was selected as the optimal. 

**Figure 10:**
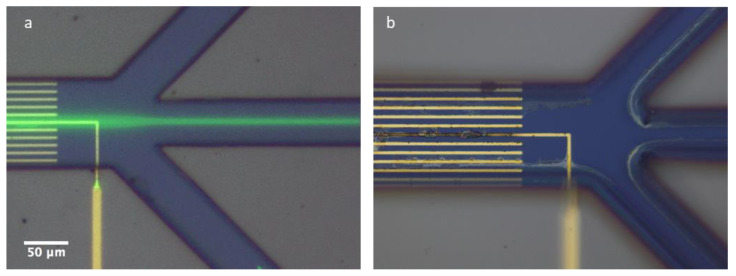
(**a**) Eighty-four-nanometer PS particles focused towards the center of the channel and directed towards the center outlet. Flow speed 50 µL/h at 2 V. (**b**) After prolonged (hours) exposure to 2 V potential, the center gold electrodes showed a significant amount of degradation/dirt accumulation.

The DC chip showed equally good alignment results. Figure 10a shows the fluorescence image superimposed on the optical image for 84 nm polymer particles that are clearly focused in the middle of the channel with a voltage of 2 V applied at the middle electrode and a flow rate of 50 µL/h. After prolonged exposure (hours) to the DC voltage, the center gold electrode showed a significant amount of degradation and/or dirt accumulation that could not readily be removed (Figure 10b). In addition, 47 nm particles were aligned under the same conditions.

### 3.4. Upconcentration Evaluation

**Figure 11:**
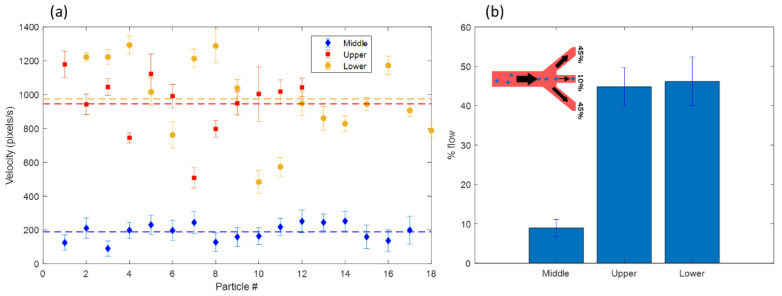
(**a**) Velocity of particles in the three outlets as calculated by ImageJ. The dotted lines indicate the average values for each outlet. (**b**) Percentage of the total flow entering each of the three outlets. The inlet shows the theoretically expected distribution.

Using ImageJ, we analyzed the velocity of up to 20 particles per outlet in order to determine the flow distribution in the three outlets. We expect that each of the side outlets should carry 45% of the flow, while the central outlet should only carry 10% of the flow. Figure 11 shows the measured velocities, calculated as described in Section 2.4. The error bars in Figure 11b are calculated based on the calculated standard deviations from the data in Figure 11a. 

The results show that the middle outlet carries 8.96% of the flow, while the two side channels carry 44.84% and 46.2%, respectively.

## 4. Discussion

Based on the optical measurements of the fabricated chips, the fabrication process can accurately reproduce the desired dimensions. The minimum distance between face-to-face electrodes was on average 30 nm larger than designed, while the minimum distance between zig-zag electrodes was on average 210 nm smaller than designed, something that only improves the chip performance, as the electric field is larger than expected for a given voltage. The DC electrode width is 240 nm larger than designed, meaning that the spacing between electrodes is smaller than expected, again resulting in higher electric fields. A certain deviation from the CAD designs is expected due to the lithography process, as process parameters such as contact mode, exposure time, resist height, and development time have an effect on the result, usually around 100–300 nm. The lithography mask itself is produced with a pixel size of 0.2 µm [18], which means that it is reasonable to expect deviations of this magnitude after fabrication. 

Due to limitations in computational power, we have simulated the particle trajectories for a channel length of 500 µm. The simulations show that the zig-zag pattern is the most effective, which is also expected, as there are more regions of a high electric field in this design compared to the face-to-face electrode design. The simulations do not take Brownian motion into consideration, something that is hard to include in COMSOL in 3D designs for a meaningful amount of particles. Instead, an algorithm was developed that performs particle tracing, with certain limitations, based on COMSOL data in a unit cell of the geometry.

The graphs (Figure 7 and Figure 8) show that even at a velocity of 25 µL/h, which is significantly higher than the originally calculated velocity of 1 µL/h, a channel length of no more than 30 mm is needed to catch the particles. Our prototype design has a channel length of 4–6 cm. The figure also shows that the end position along the channel width is independent of the flow velocity, which is expected considering the symmetric design of the electrodes across the channel length.

Our experiments were performed with 47 nm particles and show that focusing is indeed achieved well within the channel length of 4–6 mm with a flow rate of 2 µL/h. The DC chip is equally good at focusing the 47 nm particles even at higher flow rates and requires a significantly lower voltage. However, DC voltages come with a variety of issues. Particles need to be charged in order for the method to work, which is only the case at specific sample conditions (pH). Moreover, Joule heating is quite significant and can result in unwanted secondary flows that may disturb the focusing. Visual inspection of the electrodes after some hours of use shows that the electrodes are significantly degraded, possibly due to hydrolysis effects. Indeed, using voltages over 2 V immediately results in the development of bubbles, after which the electrodes show the same signs of degradation. This is not observed for the AC voltage electrodes, which is why these are preferred, even though they require a larger applied voltage. Although it is unlikely, we cannot completely exclude the possibility of electrolytic corrosion of the Ti/Au microelectrode. This happens at around the same voltages but usually at acidic or alkaline pH [19].

Apart from the DEP focusing forces, other phenomena can occur that will disturb, or maybe help, the focusing of the sub-nanometer particles. Electroosmotic fluid flow has been shown to be the dominant force in the 10 Hz–100 kHz frequency range [20], which means that it may not play a role in the presented system. Although it is not easy to predict the movement of a particle in our complicated electrode geometry, in general, electroosmosis tends to pull the particles away from the electrode edges and towards the surface of the electrodes, at least in a parallel plate electrode geometry. We would therefore expect the band of particles to broaden around the middle of the channel, more than the distance between the electrodes. As we collect particles in the middle 4 µm of the channel in the particle outlet, this effect, if at play, would not be an issue. Similarly, temperature gradients, and consequently also electrothermal flow, can occur due to the high voltage dissipated in the system, particularly if the conductivity of the fluid is high (~0.1 S/m) [20]. Again, order of magnitude calculations indicate that these forces are not significant, except when the particles are less than 1 µm from the electrodes in a highly conducting liquid. Considering the high degree of focusing that we observe in our experiments, it is likely that the used frequency of 200 kHz does not give rise to significant secondary effects.

The chip design also works as expected in terms of upconcentration. The center outlet (particle outlet) carries approximately 9% of the flow, while the two outer outlets on average carry 45.5% each. Considering the difficulties in ensuring equal pressure difference for the two outer outlet channels, the small discrepancy observed is insignificant, and in any case well within the statistical error. We note, however, that the next device iterations have a single outlet connecting the two outer channels, which ensures that they present the same hydraulic resistance to the fluid. 

**Figure 12:**
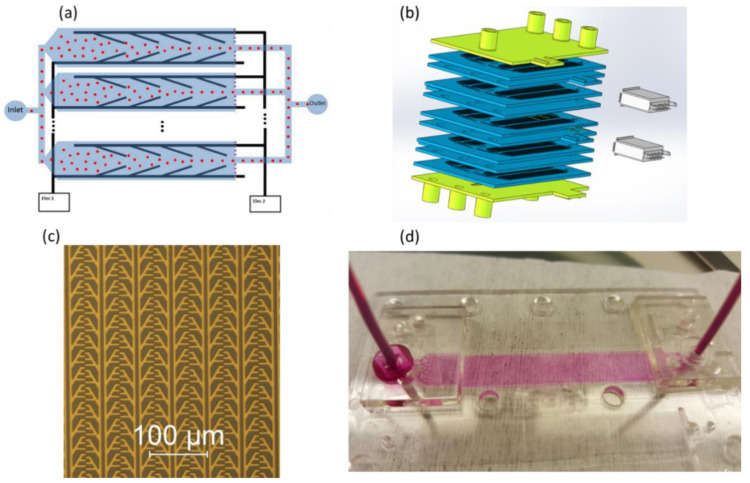
(**a**) A schematic of the parallelization concept. Only two electrodes provide voltage on all electrode pairs for an arbitrarily large number of parallel channels. All particle outlets are connected together in the chip plane, while all side outlets exit the chip from a common outlet in the bottom (not drawn). (**b**) A 3D drawing of the full chip, containing vertically stacked units, each containing 128 parallel channels. The dimensions of each chip are 65 mm × 48 mm. (**c**) Multiple electrode pairs fabricated by photolithography as described above. In this image, 6 parallel channels are shown. (**d**) A flow test of the microfluidic part of the device. For testing purposes, every 32 parallel channels were grouped together with one common inlet and outlet.

The flow rate used for the upconcentration experiments was 2 µL/h up to 50 µL/h, which is very slow. However, the electrode design can easily be adapted for massive parallelization. Figure 12 shows a schematic of the parallelization concept, along with a prototype of the multiple electrodes and 128 channels. Each layer can therefore provide a flow rate of 256 µL/h up to 6.4 mL/h, and by vertically stacking 10 such chips, we achieve a total flow rate of 2.5 mL/h up to 64 mL/h on a 35 cm^2^ footprint. Depending on the application, this can be further enhanced. However, we acknowledge that for applications requiring the processing of several liters per hour, other methods of upconcentration need to be applied beforehand.

## 5. Conclusions

In conclusion, we have presented a continuous flow upconcentration system for nanoparticles using dielectrophoresis. The prototype was able to upconcentrate particles with sizes as low as 47 nm at a flow rate of 2 µL/h. We showed how the electrode design was optimized for achieving a maximum force on the particles and how the design is scalable and can be massively parallelized in order to achieve the much higher flow rates required for most applications.

## Data Availability

The data presented in this study are available on request from the corresponding author.
